# Nigribactin, a Novel Siderophore from *Vibrio nigripulchritudo*, Modulates *Staphylococcus aureus *Virulence Gene Expression

**DOI:** 10.3390/md10112584

**Published:** 2012-11-21

**Authors:** Anita Nielsen, Maria Mansson, Matthias Wietz, Anders N. Varming, Richard K. Phipps, Thomas O. Larsen, Lone Gram, Hanne Ingmer

**Affiliations:** 1 Department of Veterinary Disease Biology, Faculty of Health and Medical Sciences, University of Copenhagen, DK-1870 Frederiksberg C, Denmark; Email: anini@sund.ku.dk (A.N.); anva@sund.ku.dk (A.N.V.); 2 Center for Microbial Biotechnology, Department of Systems Biology, Technical University of Denmark, DK-2800 Kongens Lyngby, Denmark; Email: maj@bio.dtu.dk (M.M.); xanthar@theviruz.com (R.K.P.); tol@bio.dtu.dk (T.O.L.); 3 National Food Institute, Technical University of Denmark, DK-2800 Kongens Lyngby, Denmark; Email: mwietz@ucsd.edu (M.W.); gram@food.dtu.dk (L.G.); 4 Scripps Institution of Oceanography, Center for Marine Biotechnology and Biomedicine, University of California San Diego, La Jolla, CA 92093, USA

**Keywords:** nigribactin, siderophore, *Vibrio*, *Staphylococcus aureus*, *spa*, *agr*

## Abstract

*Staphylococcus aureus* is a serious human pathogen that employs a number of virulence factors as part of its pathogenesis. The purpose of the present study was to explore marine bacteria as a source of compounds that modulate virulence gene expression in *S. aureus*. During the global marine Galathea 3 expedition, a strain collection was established comprising bacteria that express antimicrobial activity against *Vibrio anguillarum* and/or *Staphylococcus aureus*. Within this collection we searched colony material, culture supernatants, and cell extracts for virulence modulating activity showing that 68 out of 83 marine bacteria (affiliated with the *Vibrionaceae *and *Pseudoalteromonas *sp*.*) influenced expression of *S. aureus hla* encoding α-hemolysin toxin and/or *spa* encoding Protein A. The isolate that upon initial screening showed the highest degree of interference (crude ethyl acetate extract) was a *Vibrio nigripulchritudo*. Extraction, purification and structural elucidation revealed a novel siderophore, designated nigribactin, which induces *spa* transcription. The effect of nigribactin on *spa* expression is likely to be independent from its siderophore activity, as another potent siderophore, enterobactin, failed to influence *S. aureus* virulence gene expression. This study shows that marine microorganisms produce compounds with potential use in therapeutic strategies targeting virulence rather than viability of human pathogens.

## 1. Introduction

The marine environment has proven to be a reservoir of microorganisms producing compounds with interesting biomedical properties [1]. Examples of such compounds include thiopeptides from a sponge-associated *Bacillus cereus* strain with antibacterial activity against multiple drug resistant strains of staphylococci and enterococci [2]; andrimid, a broad spectrum antibiotic produced by *Vibrio coralliilyticus *[3], and the antibiotic holomycin that interferes with RNA synthesis purified from *Photobacterium halotolerans* [3]. While new antibacterial compounds may prove efficient in treating infectious diseases, human pathogens have a profound ability to acquire resistance resulting in serious health care problems. These include methicillin-resistant *Staphylococcus aureus *(MRSA), vancomycin-resistant *Enterococcus *as well as extended-spectrum cephalosporin-resistant *Escherichia coli* and *Klebsiella pneumoniae* [4,5]. To address the therapeutic failures associated with antibiotic resistance, other strategies, including anti-virulence therapies, are being considered. Antivirulence agents inhibit the production or activity of disease-causing factors of the infecting organism, and thereby disarm the pathogen of its virulence traits [6]. As quorum-sensing (QS) signalling systems are central regulators of virulence gene expression in many pathogens while being absent in humans, they represent highly promising targets for the development of anti-virulence therapeutics, possibly in combination with traditional antibiotics [7,8,9]. Several quorum sensing inhibitors (QSIs) targeting QS systems in Gram-negative pathogens have been identified, including ajoene from garlic that reduces the infective ability of *Pseudomonas aeruginosa* in a pulmonary infectious mouse model [10]. 

In Gram-positive bacteria, QS is commonly mediated by auto-inducing cyclic peptides. One example is the *agr* quorum sensing system in *Staphylococcus aureus* [11]. *S. aureus* causes a variety of infections ranging from mild skin infections to life-threatening bacteremia and endocarditis, with many strains being resistant to a number of antibiotics [12]. Pathogenesis of *S. aureus *is attributed to a multitude of virulence factors, of which a major part is controlled by *agr* [11]. The *agr* QS system is composed by an external signal, an autoinducing cyclic peptide that upon binding to the membrane-bound sensor histidine kinase (AgrC) activates the response regulator AgrA and induces virulence gene expression via a regulatory RNA, RNAIII [13,14]. Activation of *agr* results in expression of extracellular virulence factors including the key toxin, α-hemolysin, while cell surface-associated virulence factors, such as Protein A, are repressed [14]. Previously, we identified a putative QSI compound produced by the marine bacterium *Photobacterium halotolerans* that dramatically reduces *hla* and RNAIII expression while increasing *spa* production [15]. To address how abundant such compounds are in the marine environment we have screened a collection of marine bacteria for compounds that modulate *S. aureus* virulence gene expression.

## 2. Results and Discussion

### 2.1. Modulation of *S. aureus* Virulence Gene Expression by Marine Bacteria

**Figure 1:**
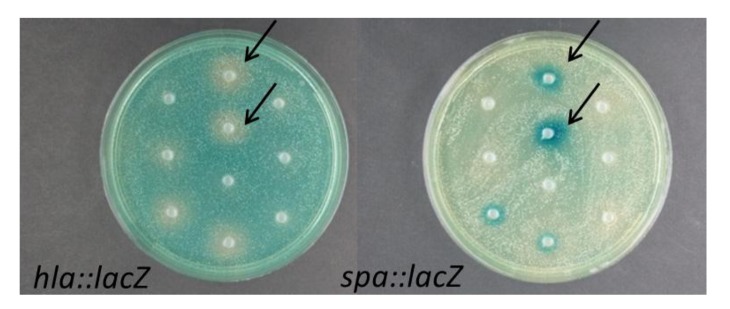
Screening of crude EtOAc extracts of *Vibrio nigripulchritudo* isolates S2600 (top arrow) and S2604 (bottom arrow) obtained from the Solomon Sea [16] in the *S. aureus hla::lacZ* and *spa::lacZ S. aureus* reporter fusion assays.

As part of the global marine Galathea 3 expedition, a collection of bacterial strains belonging to the genera *Vibrio*, *Ruegeria*, and *Pseudoalteromonas* was established based on antibacterial activity against *Vibrio anguillarum* and/or *Staphylococcus aureus* [16]. Using a reporter fusion assay [17] we screened culture extracts, supernatants, and colony material of 83 strains from this collection for the ability to inhibit expression of *S. aureus hla* (α-hemolysin) as well as interference with the *S. aureus agr* quorum sensing system reported as decreased *hla* and increased *spa* expression (Figure 1, Table 1). 

Colony material from almost all tested *Vibrionaceae* strains reduced *hla* expression, while ethylacetate (EtOAc) extracts of 17 strains and only a single culture supernatant showed this activity (Table 1). Nine extracts and colony material of 8 strains showed both repression of *hla* and induction of *spa* transcription. 

**Table 1:** Screening of marine bacterial material, extracts and culture supernatants for interference with *S. aureus* virulence gene expression. The number of isolates displaying down-regulation of *hla* and combined down-regulation of *hla* with up-regulation of *spa* are listed in the table.

Genus/family	Number of strains tested	*hla* interference			*hla*/*spa* interference		
		Colony material	Extract	Supernatant	Colony material	Extract	Supernatant
*Pseudoalteromonas*	41	37	15	0	19	0	0
*Vibrionaceae*	37	30	15	1	8	9	0
*Ruegeria*	5	0	0	0	0	0	0

Colony material of 37 out of 41 *Pseudoalteromonas* strains reduced *hla* expression, and this activity was retained in 15 of the EtOAc extracts. When *agr* interference was monitored as the combination of *hla* repression and spa induction, none of the EtOAc extracts proved positive whereas colony material from 19 strains did. The active species covered *P. phenolica*, *P. rubra*, *P. ruthenica*, and *P. luteoviolacea*. Comparison with previous work on antibiotic activity of the strains tested here [16] showed that growth inhibition is often independent from modulation of virulence gene expression (see supplementary data). None of the five tested *Ruegeria* strains affected virulence gene expression.

The present study adds to recent work of marine bacteria as sources of QS inhibitors and modulators of virulence gene expression. A marine *Bacillus* species was found to interfere with QS-controlled virulence factor production and biofilm formation in *Pseudomonas aeruginosa* PAO1 and violacein pigment production in *Chromobacterium violaceum* [18]. Ability to interfere with QS in *P. aeruginosa* was also seen in marine microorganisms isolated around the Great Barrier Reef. Of 284 tested extracts, 64 (23%) were active in a general, LuxR-derived QS screen, and of these 36 (56%) were also active in a specific *P. aeruginosa* QS screen [9]. Thus, marine bacteria seem to be common producers of compounds targeting virulence gene expression in both Gram-positive and Gram-negative bacteria possibly through modulation of QS systems. 

### 2.2. Interference of Virulence Gene Expression by *Vibrio nigripulchritudo*

**Figure 2:**
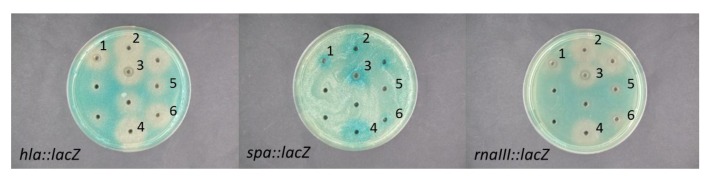
Effect of EtOAc extracts of *Vibrio nigripulchritudo* isolates (1: S2604, 2: S2600, 3: S2601, 4: S2603, 5: S1072, 6: S2156) grown stagnant with 0.4% melibiose on expression of *hla*, *spa* and *rnaIII S. aureus* reporter fusions. Clearing zones around the wells represents restricted growth and antimicrobial activity of the tested compound.

One strain, *Vibrio nigripulchritudo* S2604, displayed particularly prominent reduction of *hla* expression while increasing the expression of *spa* (Figure 1). The activity was expressed both under shaken and stagnant growth conditions. Under stagnant conditions the activity was enhanced when substituting glucose with melibiose (data not shown). In addition to *V. nigripulchritudo* S2604, we examined the five remaining isolates of *V. nigripulchritudo* grown in the presence of melibiose without aeration to determine if the ability to modulate *S. aureus* virulence gene expression was unique to strain S2604 or a general property of *V. nigripulchritudo *(Figure 2). To address whether *V. nigripulchritudo* strains directly influence *agr* quorum sensing the extracts were screened for effect on expression of the regulatory RNAIII molecule, one of the key effector molecules of the *agr* quorum sensing system [19]. Here, we observed that extracts of some *V. nigripulchritudo* strains reduced RNAIII expression whereas for other extracts the RNAIII expression was only marginally affected (Figure 2), indicating that the different *V. nigripulchritudo *strains produce a variety of QS-modulating compounds.

### 2.3. Nigribactin, a Novel Siderophore from *Vibrio nigripulchritudo* S2604, Enhances *Spa* Transcription

**Figure 3:**
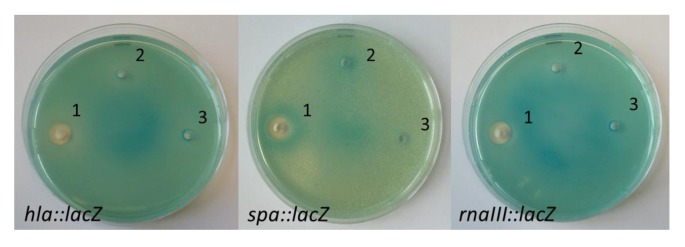
Screening of purified nigribactin for its effect on *S. aureus hla*, *spa* and RNAIII expression. 1: 2 mg·mL^−1^ nigribactin dissolved in DMSO; 2: DMSO; 3: sterile water.

Dereplication and fractionation by explorative solid-phase extraction (E-SPE) [20] of EtOAc extract obtained from *V. nigripulchritudo* S2604 indicated the presence of a novel, uncharged, apolar compound. Fractionation of a large scale S2604 extract followed by NMR revealed that a novel compound, designated nigribactin, is responsible for the *spa* enhancing activity (Figure 3). Surprisingly nigribactin did not modulate expression of *hla* and RNAIII, indicating that several compounds in the original extract influence virulence gene expression (Figure 3). 

**Figure 4:**
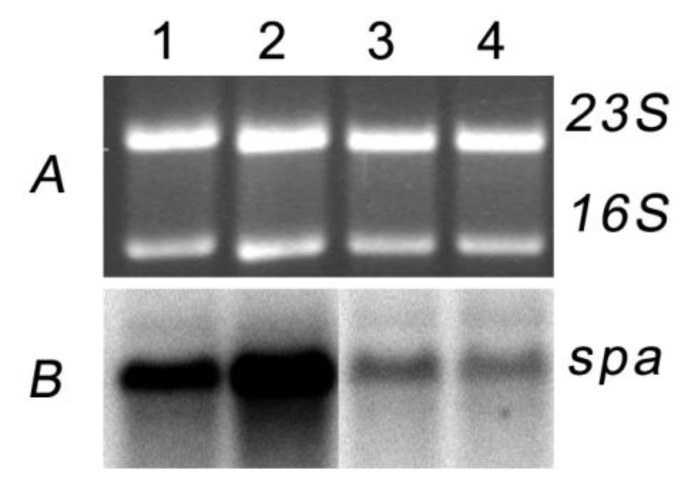
Transcription of *spa* is induced in the presence of nigribactin at low optical density but not in transition to stationary phase. Equal amounts of RNA from *S. aureus* NCTC8325-4 sampled 30 min (1, 2) and 90 min (3, 4) after addition of DMSO (1, 3) or 5µg·mL^−1^ nigribactin (2, 4) both added at OD_600_= 0.4 reacted with a *spa* specific probe.

At high concentrations nigribactin inhibits growth of *S. aureus* as observed by lack of growth closest to the well (Figure 3) with a minimal inhibitory concentration during growth in liquid medium of >10 μg·mL^−1^ (data not shown). However, in the plate assay (Figure 3) the *spa*-inducing activity was observed further from the well where only sub-lethal concentrations of nigribactin are present. The ability of nigribactin to enhance *spa* transcription was confirmed by Northern blot analysis showing a substantial increase in *spa* transcription in the exponential growth phase (Figure 4).

**Figure 5:**
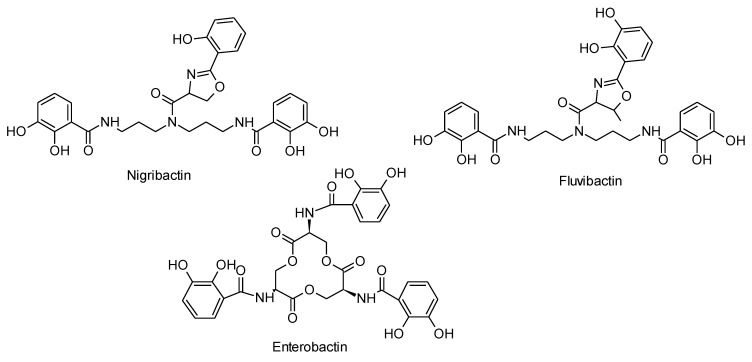
Structures of the bacterial siderophores nigribactin (this study), fluvibactin [21] and enterobactin [23].

Nigribactin (C_30_H_32_N_4_O_9_, calc monoisotopic mass 592.2169 Da) showed to be a catechol hydroxyphenyloxalone with a norspermidine backbone, giving it high structural similarity to siderophores from *Vibrio* such as vibriobactin and fluvibactin [21]. Siderophores are low molecular weight iron chelators typically produced in response to low-iron stress [22]. The structure of nigribactin was established by comparison of 1D and 2D NMR data recorded in DMSO-*d*_6_ (^13^C data given in Section 3.3) to data for fluvibactin from *Vibrio fluvialis* [21]. Analysis of the NMR data revealed that the nigribactin structure differs from that of fluvibactin only by containing one less methyl group in the 5-membered oxazoline ring and one less hydroxyl group (Figure 5).

**Figure 6:**
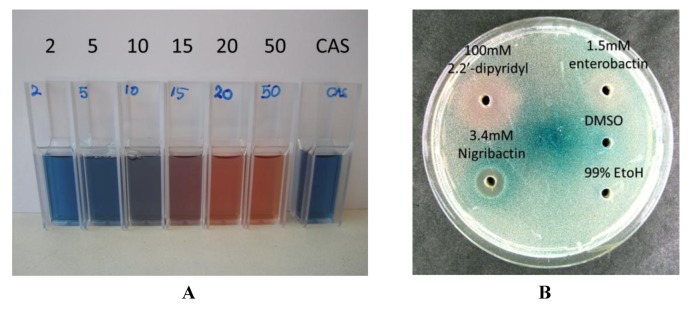
(**A**) Confirmation of siderophore activity of nigribactin by addition at 2, 5, 10, 15, 20, and 50 µM to vials carrying Chrome azurol S (CAS);(**B**) Test of enterobactin (dissolved in DMSO) and 2,2′-dipyridyl (dissolved in 99% EtOH) against the *S. aureus *8325-4 *spa::lacZ* reporter fusion [26] incorporated into an agar plate.

The structural similarity of nigribactin to known siderophores prompted us to address if nigribactin is a siderophore. We confirmed prominent iron-chelating activity of nigribactin by examining dilutions of purified nigribactin using the colometric CAS assay [24] (Figure 6A). However, the siderophore activity of nigribactin appears not to be responsible for the effect on *spa* expression as neither another catechol siderophore, enterobactin [25] (Figure 5), nor 2,2-dipyridyl, an iron chelating compound, induced *spa* transcription (Figure 6B).

## 3. Experimental Section

### 3.1. Bacterial Strains

Of 512 marine bacterial strains isolated during the global Galathea 3 expedition [16], 83 strains were selected for the present study. The screening assay applied in this study is described by [17] using *S. aureus* strains carrying different gene reporter fusions, including *S. aureus* 8325-4 *hla*::*lacZ* [26], *S. aureus* 8325-4 *spa*::*lacZ* [26] and *S. aureus* 8325-4 *rnaIII*::*lacZ* [27,28]. *S. aureus* strain 8325-4 [29] was used for Northern blot analyses.

### 3.2. Bacterial Growth Conditions, Culture Extraction and Virulence Gene Expression Assay

Marine bacteria were grown in 30 mL sea salt solution (SSS; Sigma S9883; 40 g·L^−1^) with 0.4% glucose and 0.3% casamino acids for three days at 25 °C with (200 rpm) and without (0 rpm) aeration. Culture supernatants were prepared by sterile filtration. Cultures were extracted with an equal volume of EtOAc, transferring the organic phase to a new vial, and evaporating under nitrogen gas until dryness. Fractionation by explorative solid-phase extraction (E-SPE) was performed according to [20]. Dry extracts and fractions were redissolved in 300 µL 80% EtOH for biological testing as described in [17]. For screening of colony material, marine bacteria were grown on Marine Agar 2216 (Difco 212185) for 24 h at 25 °C, and a lump of colony material was placed on top of agar plates containing *S. aureus* [17] but without wells in the plates, and incubated for 48 h at 30 °C. By using a combination of reporter strains looking for both up- and -down regulation, we were able to detect and exclude strains being natural producers of β-galactosidase.

### 3.3. Northern Blot Analysis

*S. aureus* 8325-4 was grown in TSB at 37 °C at 200 rpm. Nigribactin was added at OD_600_ = 0.4 and samples for RNA extraction were taken after 30 and 90 min. Northern blot analysis using a probe targeting *spa* was performed as described previously [30]. Probes were made using the primers *spa* forward (5′-GGG GGT GTA GGT ATT GCA TCT G-3′) and *spa* reverse (5′-GGG GCT CCT GAA GGA TCG TC-3′).

### 3.4. Purification and Structural Elucidation of Nigribactin

Strain S2604 was grown in 2 L sea salt solution (Sigma S9883; 40 g·L^−1^) with 0.4% melibiose and 0.3% casamino acids for three days (0 rpm) at 25 °C. On day 3, the culture was extracted with 750 mL EtOAc for 24 h. The organic extract was dry loaded onto 10 g Sepra ZT C18 (Phenomenex, Torrance, CA) and dried before packing into a 60 g SNAP column (Biotage, Uppsala, Sweden) with 50 g pure resin in the base. Using an Isolera flash purification system (Biotage) the extract was subjected to a crude fractionation using an acetonitrile (MeCN)/H_2_O gradient (flow rate 40 mL·min^−1^) starting with 10% MeCN (2 column volumes (CV), isocratic), increasing to 100% MeCN (10 CV) before washing with 100% MeCN (2 CV). Fractions were automatically collected using UV detection (210 and 320 nm). The fractions inducing *spa *activity (120 mg) were pooled, evaporated, and redissolved in 1.2 mL EtOAc/methanol (MeOH; 1:3 v/v) for diol separation (Isolute diol, Biotage) on the Isolera system. A total of nine fractions (fraction size 12 mL) were collected from the diol column (10 g SNAP column) ranging from heptane, dichloromethane (DCM), EtOAc to pure MeOH, running under gravity. The fractions (28 mg total) with *spa *activity (25% DCM in heptane to 100% MeOH) were pooled and purified on a Luna II C_18_ column (250 × 10 mm, 5 μm) (Phenomenex) using a 45%–70% MeCN/H_2_O gradient (buffered with 20 mM formic acid, flow rate 4 mL·min^−1^) over 20 min on a Gilson 322 liquid chromatograph with a 215 liquid handler/injector (BioLab, Risskov, Denmark). All fractions were analysed by LC-UV-MS according to standard procedures [20] before pooling. This yielded 1.6 mg of nigribactin. 

**Figure 7:**
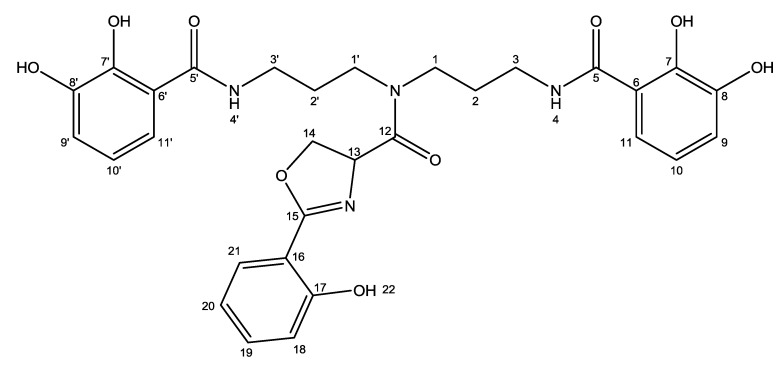
Structure of nigribactin with numbered atoms as assigned in Table 2.

NMR spectra were recorded on a Varian Unity Inova 500 MHz spectrometer equipped with a 5 mm probe using standard pulse sequences. ^13^C data was confirmed on a Bruker Avance 800 MHz spectrometer at the Danish Instrument Center for NMR Spectroscopy of Biological Macromolecules. The NMR data used for the structural assignment of nigribactin (Figure 7) were acquired in DMSO-*d*_6_ (Table 2).

**Table 2:** NMR spectroscopic data (DMSO-*d*_6_) of nigribactin.

Atom	*δ*_C_ (ppm)	*δ*_H_ (ppm) (multiplicity, *J* (Hz))	HMBC
1a	43.2	3.38 (1H, m)	1′, 2, 3, 12
1b	43.2	3.36 (1H, m)	1′, 2, 3, 12
2	26.8	1.77 (2H, m)	-
3	36.3	3.26 (2H, m)	1, 2, 5
4	-	8.74	5
5	169.4	-	-
6	114.8	-	-
7	149.4	-	-
8	145.9	-	-
9	118.6	6.88 (1H, d, 7.6)	7, 8, 11
10	117.7	6.65 (1H, t, 7.6)	6, 8
11	116.9	7.22 (1H, d, 8.0)	5, 7, 9
1′a	44.6	3.68 (1H, m)	2′, 12
1′b	44.6	3.56 (1H, m)	2′, 12
2′	28.2	1.95 (2H, p, 7.2)	1′, 3′
3′	36.3	3.38 (2H, m)	1′, 2′, 5′
4′	-	8.83	5′
5′	169.6	-	-
6′	114.8	-	-
7′	149.4	-	-
8′	145.9	-	-
9′	118.6	6.88 (1H, d, 7.6)	7′, 8′, 11′
10′	117.7	6.65 (1H, t, 7.6)	6′, 8′
11′	116.9	7.26 (1H, d, 8.0)	5′, 7′, 9′
12	168.3	-	-
13	64.2	5.36 (dd, 9.5, 6.7)	12, 15
14a	69.2	4.77 (1H, t, 7.5)	12, 13, 15
14b	69.2	4.54 (1H, t, 8.9)	12, 15
15	165.3	-	-
16	109.6	-	-
17	158.6	-	-
18	116.4	6.97 (1H, d, 8.3)	16, 17, 20
19	133.9	7.44 (1H, t, 7.9)	17, 21
20	118.9	6.93 (1H, t, 7.6)	16, 18
21	127.9	7.62 (1H, dd, 7.8, 1.1)	17, 19
22 (–OH)	-	11.7	16, 17, 18
–OH	-	12.7	-
–OH	-	12.6	-
–OH	-	9.12	-
–OH	-	9.09	-

## 4. Conclusions

This study shows that a substantial number of marine bacteria (80%), collected from various marine habitats worldwide, are able to influence *S. aureus* virulence gene expression. From *Vibrio nigripulchritudo* we isolated a new siderophore, nigribactin, which enhances the expression of *spa* encoding Protein A. While the crude extract of this bacterium also showed *hla*-repressing activity, we failed to isolate a single compound both repressing *hla* and inducing *spa* expression. However, since a large number of strains displayed this combination of activities such compounds are likely to be abundant. From a biological perspective, it is intriguing that bacteria from marine habitats produce compounds that influence virulence gene expression of a pathogen normally associated with warm-blooded animals. Thus, our study shows that marine bacteria are a source of compounds that affect virulence gene expression in *S. aureus* and ultimately, such compounds may aid in the treatment of infectious diseases.

## Supplementary Files

Supplementary File 1: Supplementary Information (PDF, 195 KB)
