# Supplementary material for: Nigribactin, a Novel Siderophore from Vibrio nigripulchritudo, Modulates Staphylococcus aureus Virulence Gene Expression
Source: Mar Drugs. 2012 Nov 21;10(11):2584–95. doi: 10.3390/md10112584 (PMC3509537; doi:10.3390/md10112584)
Supplement: Supplementary File 1: — Supplementary Information (PDF, 195 KB) [file marinedrugs-10-02584-s001.pdf]

Supplementary Information

Table S1. Screening of marine bacterial material, extracts and culture supernatants for interference with *S. aureus* virulence gene expression. Down-Regulation of *hla* and combined down-regulation of *hla* with up-regulation of *spa* is listed in the table. Data on genus/species identification and antimicrobial activity are from [1].

**Isolate No.**	**Genus**	***hla* interference (*hla* down)**			***hla*/*spa* interference (*hla* down, *spa* up)**			**Antimicrobial activity against *S. aureus***
		**Supernatant**	**Extract**	**Colony mass**	**Supernatant**	**Extract**	**Colony mass**	
S0788	*Vibrio* sp. (*coralliilyticus*)	-	-	+	-	-	-	
S0842	*Vibrio* sp.	-	-	+	-	-	-	-
S1072	*Vibrio* sp. (*nigripulchritudo*)	-	+	+	-	-	-	-
S1078	*Vibrio* sp. (*harveyi*)	-	-	+	-	-	-	-
S1124	*Vibrio* sp.	-	+	+	-	-	-	++
S1162	*Vibrio* sp. (*fluvialis*)	-	+	-	-	-	-	-
S1175	*Vibrio* sp.	-	-	+	-	-	-	++
S1346	*Vibrio* sp. (*neptunius*)	-	+	+	-	-	-	++
S1348	*Vibrio* sp. (*parahaemolyticus*)	-	-	-	-	-	-	-
S1368	*Vibrio* sp. (*harveyi*)	-	-	-	-	-	-	+
S1396	*Vibrio* sp.	-	-	-	-	-	-	++
S1614	*Vibrio* sp. (*coralliilyticus*)	-	+	+	-	+	-	-
S2052	*Vibrio* sp.	+	+	+	-	-	-	+++
S2056	*Vibrio* sp. (*coralliilyticus*)	-	-	+	-	-	-	++
S2150	*Vibrio* sp.	-	-	+	-	-	-	++
S2156	*Vibrio* sp. (*nigripulchritudo*)	-	+	+	-	+	+	-
S2320	*Vibrio* sp.	-	-	+	-	-	-	+
S2322	*Vibrio* sp.	-	-	-	-	-	-	-
S2394	*Vibrio* sp.	-	-	+	-	-	-	-
S2407	*Vibrio* sp.	-	+	+	-	+	-	+
S2536	*Vibrio* sp. (*harveyi*)	-	-	+	-	-	-	+
S2537	*Vibrio* sp. (*penaeicida*)	-	+	+	-	+	+	+
S2600	*Vibrio* sp. (*nigripulchritudo*)	-	+	+	-	+	+	+++

Table S1. *Cont.*

S2601	*Vibrio* sp. (*nigripulchritudo*)	-	+	+	-	+	+	-
S2603	*Vibrio sp.* (*nigripulchritudo*)	-	+	+	-	+	+	++
S2604	*Vibrio sp.* (*nigripulchritudo*)	-	+	+	-	+	+	-
S2687	*Vibrio sp.* (*parahaemolyticus*)	-	-	+	-	-	+	+
S2718	*Vibrio sp.*	-	-	+	-	-	+	++
S2726	*Vibrio sp.*	-	-	+	-	-	-	+
S3926	*Vibrio* sp. (*neptunius*)	-	-	+	-	-	-	+++
S4051	*Vibrio* sp.	-	-	+	-	-	-	+++
S4053	*Vibrio* sp.	-	-	+	-	-	-	++
S4073	*Vibrio* sp.	-	+	-	-	+	-	+++
S4074	*Vibrio* sp. (*chagasii*)	-	-	+	-	-	-	+
S4077	*Vibrio* sp.	-	-	+	-	-	-	++
S4202	*Vibrio* sp.	-	-	-	-	-	-	-
S4225	*Vibrio* sp.	-	+	+	-	-	-	+
S1358	*Ruegeria* sp. (*mobilis*/*pelagia*)	-	-	-	-	-	-	+++
S1611	*Ruegeria* sp. (*mobilis*/*pelagia*)	-	-	-	-	-	-	+++
S2145	*Ruegeria* sp. (*mobilis*/*pelagia*)	-	-	-	-	-	-	+
S1942	*Ruegeria* sp. (*mobilis*/*pelagia*)	-	-	-	-	-	-	+++
S2405	*Ruegeria* sp. (*mobilis*/*pelagia*)	-	-	-	-	-	-	++
S0185	*Pseudoalteromonas marina*	-	-	+	-	-	-	-
S0201	*Pseudoalteromonas* sp.	-	+	+	-	-	-	-
S0327	*Pseudoalteromonas* sp.	-	-	+	-	-	+	-
S0410	*Pseudoalteromonas marina*	-	-	+	-	-	-	-
S0554	*Pseudoalteromonas marina*	-	+	+	-	-	-	-
S0577	*Glaciecola* sp. (*Pseudoalteromonas* sp.)	-	+	+	-	-	-	-
S1189	*Pseudoalteromonas* sp. (*phenolica*)	-	-	+	-	-	+	+++
S1608	*Pseudoalteromonas* sp.	-	-	+	-	-	-	-
S1609	*Pseudoalteromonas* sp.	-	-	+	-	-	-	-

Table S1. *Cont.*

S1612	*Pseudoalteromonas* sp.	-	+	+	-	-	-	-
S1650	*Pseudoalteromonas* sp.	-	-	+	-	-	-	-
S1690	*Pseudoalteromonas* sp.	-	-	+	-	-	-	-
S1845	*Pseudoalteromonas piscicida*	-	-	+	-	-	-	++
S1925	*Pseudoalteromonas piscicida*	-	-	+	-	-	-	++
S1943	*Pseudoalteromonas rubra*	-	+	+	-	-	+	+++
S1946	*Pseudoalteromonas rubra*	-	+	+	-	-	+	+++
S2049	*Pseudoalteromonas piscicida*	-	-	+	-	-	-	++
S2050	*Pseudoalteromonas piscicida*	-	+	+	-	-	-	++
S2053	*Pseudoalteromonas piscicida*	-	-	+	-	-	-	++
S2231	*Pseudoalteromonas* sp.	-	+	+	-	-	+	+
S2233	*Pseudoalteromonas* sp.	-	+	-	-	-	-	+
S2471	*Pseudoalteromonas rubra*	-	+	+	-	-	+	++
S2472	*Pseudoalteromonas rubra*	-	-	-	-	-	-	-
S2599	*Pseudoalteromonas rubra*	-	-	+	-	-	+	++
S2607	*Pseudoalteromonas luteoviolacea*	-	-	-	-	-	-	+++
S2676	*Pseudoalteromonas rubra*	-	-	+	-	-	+	++
S2717	*Pseudoalteromonas ruthenica*	-	-	+	-	-	+	+
S2893	*Pseudoalteromonas* sp.	-	-	+	-	-	+	-
S2899	*Pseudoalteromonas ruthenica*	-	+	+	-	-	+	++++
S3137	*Pseudoalteromonas ruthenica*	-	+	+	-	-	+	++++
S3260	*Pseudoalteromonas* sp.	-	-	+	-	-	+	-
S3431	*Pseudoalteromonas* sp.	-	+	+	-	-	-	-
S3655	*Pseudoalteromonas spongiae*	-	+	+	-	-	-	-
S3665	*Pseudoalteromonas* sp.	-	-	+	-	-	+	+++
S3673	*Pseudoalteromonas* sp.	-	-	+	-	-	+	++
S3788	*Pseudoalteromonas* sp.	-	-	-	-	-	-	-
S4052	*Pseudoalteromonas* sp. (*phenolica*)	-	-	+	-	-	+	+++
S4054	*Pseudoalteromonas luteoviolacea*	-	-	+	-	-	+	++++

Table S1. *Cont.*

S4055	*Pseudoalteromonas* sp.	-	+	+	-	-	+	+++
S4059	*Pseudoalteromonas rubra*	-	-	+	-	-	+	+++
S4382	*Pseudoalteromonas ruthenica*	-	-	+	-	-	-	++++
S1612	*Pseudoalteromonas* sp.	-	+	+	-	-	-	-
S1650	*Pseudoalteromonas* sp.	-	-	+	-	-	-	-
S1690	*Pseudoalteromonas* sp.	-	-	+	-	-	-	-
S1845	*Pseudoalteromonas piscicida*	-	-	+	-	-	-	++
S1925	*Pseudoalteromonas piscicida*	-	-	+	-	-	-	++
S1943	*Pseudoalteromonas rubra*	-	+	+	-	-	+	+++
S1946	*Pseudoalteromonas rubra*	-	+	+	-	-	+	+++
S2049	*Pseudoalteromonas piscicida*	-	-	+	-	-	-	++
S2050	*Pseudoalteromonas piscicida*	-	+	+	-	-	-	++
S2053	*Pseudoalteromonas piscicida*	-	-	+	-	-	-	++
S2231	*Pseudoalteromonas* sp.	-	+	+	-	-	+	+
S2233	*Pseudoalteromonas* sp.	-	+	-	-	-	-	+
S2471	*Pseudoalteromonas rubra*	-	+	+	-	-	+	++
S2472	*Pseudoalteromonas rubra*	-	-	-	-	-	-	-
S2599	*Pseudoalteromonas rubra*	-	-	+	-	-	+	++
S2607	*Pseudoalteromonas luteoviolacea*	-	-	-	-	-	-	+++
S2676	*Pseudoalteromonas rubra*	-	-	+	-	-	+	++
S2717	*Pseudoalteromonas ruthenica*	-	-	+	-	-	+	+
S2893	*Pseudoalteromonas* sp.	-	-	+	-	-	+	-
S2899	*Pseudoalteromonas ruthenica*	-	+	+	-	-	+	++++
S3137	*Pseudoalteromonas ruthenica*	-	+	+	-	-	+	++++
S3260	*Pseudoalteromonas* sp.	-	-	+	-	-	+	-
S3431	*Pseudoalteromonas* sp.	-	+	+	-	-	-	-
S3655	*Pseudoalteromonas spongiae*	-	+	+	-	-	-	-
S3665	*Pseudoalteromonas* sp.	-	-	+	-	-	+	+++
S3673	*Pseudoalteromonas* sp.	-	-	+	-	-	+	++

Table S1. *Cont.*

S3788	*Pseudoalteromonas* sp.	-	-	-	-	-	-	-
S4052	*Pseudoalteromonas* sp. (*phenolica*)	-	-	+	-	-	+	+++
S4054	*Pseudoalteromonas luteoviolacea*	-	-	+	-	-	+	++++
S4055	*Pseudoalteromonas* sp.	-	+	+	-	-	+	+++
S4059	*Pseudoalteromonas rubra*	-	-	+	-	-	+	+++
S4382	*Pseudoalteromonas ruthenica*	-	-	+	-	-	-	++++

References

1. Gram, L.; Melchiorsen, J.; Bruhn, J.B. Antibacterial activity of marine culturable bacteria collected from a global sampling of ocean surface waters and surface swabs of marine organisms. *Mar. Biotechnol.* **2010**, *12*, 439–451.

© 2012 by the authors; licensee MDPI, Basel, Switzerland. This article is an open access article distributed under the terms and conditions of the Creative Commons Attribution license (http://creativecommons.org/licenses/by/3.0/).
